# Preparation of γ-Divinyl-3-Aminopropyltriethoxysilane Modified Lignin and Its Application in Flame Retardant Poly(lactic acid)

**DOI:** 10.3390/ma11091505

**Published:** 2018-08-22

**Authors:** Yan Song, Xu Zong, Nan Wang, Ning Yan, Xueying Shan, Jinchun Li

**Affiliations:** 1 Faculty of Materials Science & Engineering, Changzhou University, Changzhou 213164, Jiangsu, China; ysong@cczu.edu.cn (Y.S.); 15251930097@163.com (X.Z.); wnan0824@163.com (N.W.); 2 Jiangsu Key Laboratory of Environmentally Friendly Polymer Materials, Changzhou University, Changzhou 213164, Jiangsu, China; 3 Faculty of Forestry, University of Toronto, 33 Willcocks Street, Toronto, ON M5S 3B3, Canada; ning.yan@utoronto.ca; 4 School of Environmental and Safety Engineering, Changzhou University, Changzhou 213164, Jiangsu, China; xyshan@cczu.edu.cn

**Keywords:** wheat straw soda lignin, silicon and nitrogen elements, poly(lactic acid), thermal stability, combustion behavior

## Abstract

Lignin can be a candidate as a charring agent applied in halogen-free flame retardant polymers, and incorporation of silicon and nitrogen elements in lignin can benefit to enhancing its thermal stability and charring ability. In the present work, wheat straw alkali lignin (Lig) was modified to incorporate silicon and nitrogen elements by γ-divinyl-3-aminopropyltriethoxysilane, and the modified lignin (CLig) was combined with ammonium polyphosphate (APP) as intumescent flame retardant to be applied in poly(Lactic acid) (PLA). The flame retardancy, combustion behavior and thermal stability of PLA composites were studied by the limited oxygen index (LOI), vertical burning testing (UL-94), cone calorimetry testing (CCT) and thermogravimetric analysis (TGA), respectively. The results showed a significant synergistic effect between CLig and APP in flame retarded PLA (PLA/APP/CLig) occured, and the PLA/APP/CLig had better flame retardancy. CCT data analysis revealed that CLig and APP largely reduced the peak heat release rate (PHRR) and total heat release rate (THR) of PLA, indicating their effectiveness in decreasing the combustion of PLA. TGA results exhibited that APP and CLig improved the thermal stability of PLA at high temperature. The analysis of morphology and structure of residual char indicated that a continuous, compact and intumescent char layer on the material surface formed during firing, and had higher graphitization degree. Mechanical properties data showed that PLA/APP/CLig had higher tensile strength as well as elongation at break.

## 1. Introduction

In recent years, biodegradable polymeric materials have been paid more and more attention because of shortage of petroleum source and environmental pollution caused by non-degradable petroleum-based plastics [1,2]. Among these biopolymers, poly(lactic acid) (PLA) plays a significant role due to its raw material, lactic acid, derived from renewable resources such as corn or sugar beet, as well as its good thermal stability and great biocompatibility [3,4,5]. Meanwhile, PLA is increasingly used as a candidate for petroleum-based commodity polymers in packaging materials, textile, electronic fields, and is one of the most suitable candidate for 3D printing manufacts [6,7]. However, its easy combustibility, especially the serious dripping during burning restrains its applications in new areas such as electronics and automotive [8,9].

A promising way to give flammable materials a fire protection is to incorporate intumescent flame retardant (IFR). IFR is regarded as an effective and environment-friendly flame retardant because of its low smoke and nontoxic gases during combustion. The IFR system usually consists of three components, namely an acid source, a carbonization agent and a blowing agent. Traditional IFR system is generally composed of ammonium polyphosphate (APP) as both acid source and blowing agent, and pentaerythritol (PER) as charring agent, which has been widely applied in polyolefin materials. Nevertheless, there are some limitations for PER applied in engineering polymeric materials, such as higher humidity and easy migration to the surface of material causing the deterioration of the material’s properties. In order to deal with the above problems, many efforts have been done to search for new efficient carbon sources [1]. Additionaly, renewed interest is emerging for the sustainable development of flame retardants for polymeric materials [10]. Therefore, some biobased charring agents have been paid much more attention, such as chitosan [11], β-cyclodextrin [12,13], starch [14,15], polyhedral oligomeric silsesquioxane (POSS) [16] or lignin [17].

Lignin is an abundant, renewable, inexpensive and readily available natural macromolecule with three-dimensional network structure, and made up of three phenylpropanoid units including p-hydroxyphenyl propane, guaiacylpropane and syringylpropane units [18,19]. Moreover, lignin has been used as charring agent in intumescent flame retardant polymeric materials thanks to its containing a lot of hydroxyl groups and aromatic structure [20,21]. Reti et al. [14] prepared flame retardant PLA by incorporating lignin and APP as IFR with 40% loading amount. Moreover, kraft lignin was combined with APP to be applied in PLA texture, and it was found that the flame retardant performance of PLA texture was better than that of PLA with kraft lignin (LK) or APP alone, which could be ascribed to the synergistic effect between kraft lignin and APP [22]. It was reported that incorporation of flame retardant elements, such as phosphorus, nitrogen, silicon and so on, by chemical modification could enhance the flame retardant role of lignin in PLA. Lucie et al. [17] prepared phosphorus and nitrogen-containing lignin by two-step chemical modification, and found that compared with unmodified lignin, the modified lignin was much more effective in reducing the flammability of PLA composites as well as maintaining the time to ignition (TTI) and thermal stability of PLA. Zhang’s research group [23,24] obtained urea-modified lignin by Mannich reaction, and combined the modified lignin with APP to prepare flame retardant PLA composites. They found that the flame retardant PLA emerged much better flame retardancy and thermal stability compared with PLA containing unmodified lignin and APP. Furthermore, they prepared lignin-silica hybrids (LSH) by a sol-gel method to be applied in PLA. However, there are few reports on modification of lignin by silane to incorporate silicon and nitrogen applied in flame retardant polymeric materials.

In this paper, wheat straw alkali lignin was modified to incorporate silicon and nitrogen elements in by γ-divinyl-3-aminopropyltriethoxysilane, and the modified lignin was combined with ammonium polyphosphate (APP) as intumescent flame retardant to be applied in poly(Lactic acid)(PLA). The flame retardancy, combustion behavior and thermal stability of PLA composites were investigated by LOI, UL-94 testing, CCT and TGA, respectively. SEM and Ramman spectrum were used to characterize morphology and structure of residues after CCT. Mechanical properties were also determined.

## 2. Materials and Methods 

### 2.1. Materials

Poly(lactic acid) (PLA) (4032D) was purchased from Nature works Company, Blair, NE, USA; wheat straw soda lignin was provided by Shandong Yanghai Material Company, Jinan, China; Ammonium polyphosphate (II) (APP) was offered by Shenzhen Anzheng Chemicals Company, Shenzhen, China; silane coupling agent B201, namely γ-divinyl-3-aminopropyltriethoxysilane was supplied by Shanghai Beihe Chemicals Company, Shanghai, China. 

### 2.2. Preparation 

#### 2.2.1. Preparation of Modified Lignin by Silane Coupling Agent

**Scheme 1:**
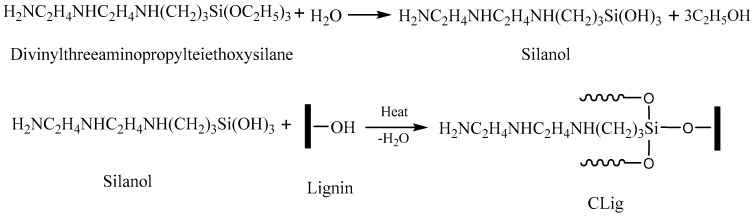
Schematic of the reaction between B201 and lignin.

Modification of lignin by γ-divinyl-3-aminopropyltriethoxysilane (Shanghai Beihe Chemicals Company, Shanghai, China) (B201) was carried out as follows: 0.5000 g B201 was dissolved into 50 mL water-ethanol (Sinapharm Chemical Reagent Co., Ltd., Shanghai, China) mixture (volume ratio of water and ethanol with 5/95) to make the B201 solutions. The solution was slowly transferred into 10 g dried lignin powder accompanied with continuous stirring. Finally, the crude product was dried at 105 °C under electric heat oven (Shanghai Huitai Instrument Manufacturing Co., Ltd., Shanghai, China) for 12 h to obtain the modified lignin (CLig). The schematic route for the reaction between silane coupling agent B201 and lignin was shown in Scheme 1.

#### 2.2.2. Preparation of PLA composites

PLA and flame retardants were respectively dried in vacuum oven (Shanghai Huitai Instrument Manufacturing Co., Ltd., Shanghai, China) at 80 °C 48 h before use. PLA composites were prepared using a SU-70B Internal mixer (Changzhou Suyan Technology Co., Ltd., Changzhou, China) at 180 °C with the roller speed 30 rpm for 10 min. The formulation of the samples was seen in Table 1. APP and CLig (or lignin) maintaining 23 wt% total loadings with different ratios were incorporated into PLA to obtain the optimum IFR system. After mixing, all the samples were hot-pressed under 10 MPa for 5 min at 180 °C to make specimens for UL-94 vertical testing, limiting oxygen index (LOI), cone calorimeter tests (CCT) and the mechanical property.

### 2.3. Characterization

#### 2.3.1. Fourier Transform Infrared Spectroscopy (FTIR)

The Avatar370 type Fourier infrared spectrometer (Nicolet Co, Ltd., Madison, WI, USA) was applied in this work. The samples were obtained by grounding a mixture of 2 mg Lig or CLig and 200 mg KBr (Shanghai Aladdin Biochemical Technology Co., Ltd., Shanghai, China) before being pressed at 10 MPa for 3 min. The scanning scope was 4000 cm^−1^~400 cm^−1^ with 4 cm^−1^ of the resolution ratio.

#### 2.3.2. Thermogravimetric Analysis (TGA)

Thermal stability testing of the samples were performed on TAQ50 TGA (WATERS Company, Milford, MA, USA) instrument at a heating rate of 20 °C/min from 50 °C to 800 °C, in which CLig was tested under air, while the PLA and flame retardant PLA composites under N_2_ atmosphere, and gas flow rate was 60 mL min^−1^. Samples were measured in a platinum crucible (Shanghai Kaizheng Instrument Co., Ltd., Shanghai, China) with a weight of about 7 mg.

#### 2.3.3. Flame Retardancy and Combustion Behavior

The flame retardancy and combustion behavior of the samples were characterized by limiting oxygen index (LOI), UL-94 vertical testing and cone calorimeter tests (CCT), respectively. Limiting oxygen index (LOI) tests were done with a JF-3 oxygen index test instrument (Jiangning County Analytical Instrument Factory, Jiangning, China) according to American Society for Testing Materials (ASTM) D2863-97 with the dimension size of 130 mm × 6.5 mm × 3.2 mm. UL-94 vertical burning level were conducted on an CTF-2 instrument (Jiangning County Analytical Instrument Factory, Jiangning, China) with sample dimensions according to American Society for Testing Materials (ASTM) D3801, and the dimension of samples is 130 mm × 13 mm × 3.2 mm. The cone calorimeter tests were tested by a Fire Testing Technology Limited cone calorimeter (East Grinstead, London, UK) following the procedures in International Organization for Standardization (ISO) 5660. All samples with dimensions of 100 mm × 100 mm × 3 mm were exposed horizontally to an external heat flux of 35 kW/m^2^.

#### 2.3.4. Characterization of Residual Chars after Limiting Oxygen Index Test

The morphology of the residual chars, obtained from the samples after limiting oxygen index testing, was observed by a Carl Zeiss (SUPRA 55) SEM (Carl Zeiss Company, Jena, Germany) instrument with an acceleration voltage of 15 kV at working distance ranging from 10 to 15 mm. Meanwhile, the residual chars’ structure was characterized by a Raman microspectrometer (Renishaw Company, London, UK) with excitation by a 514.5 nm helium-neon laser with scanning range of 100–3500 cm^−1^ at room temperature.

#### 2.3.5. Mechanical Properties

The mechanical properties of PLA and flame retardant PLA composites were measured by a CMT5504 Electronic Universal Testing Machine (MTS Industrial System (China) Co., Ltd., Shengzhen, China) at crosshead speed of 10mm/min. The dumbbell-shaped specimens of the PLA/IFR composites used for tensile testing were prepared by YF-8017 Flat Vulcanizing Machine under the pressure of 10 MPa (Yuanfeng Testing Machinery Factory, Yangzhou, China).

## 3. Results and Discussion

### 3.1. Characterization of Lig and CLig

#### 3.1.1. FTIR Analysis

**Figure 1:**
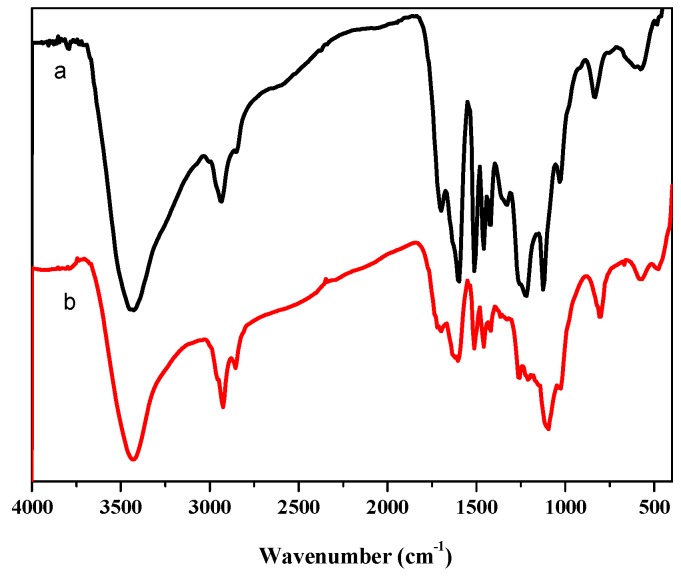
FTIR spectra of Lig (a) and CLig (b).

FTIR spectra of the lignin (Lig) and modified lignin (CLig) are shown in Figure 1 Besides the common characteristic absorption peaks of –OH group (3425 cm^−1^), –CH_2_– and –CH_3_ groups (2937 and 2852 cm^−1^), and aromatic ring (1600, 1510 and 1459 cm^−1^), there are two new absorption peaks appearing in CLig, namely, 1097 cm^−1^ caused by the stretching vibration of –Si–O–Si– group, and 805 cm^−1^ ascribed to the bending vibration [25,26]. This may be attributed to the reaction between silanols generated by the hydrolysis of γ-divinyl-3-aminopropyltriethoxysilane and reactive hydroxyl groups in lignin. Therefore, it could be inferred that silicon and nitrogen elements are incorporated into lignin, and the lignin is successfully modified by γ-divinyl-3-aminopropyltriethoxysilane.

#### 3.1.2. X-ray Fluorescence Spectroscopy Analysis

The ordinary techniques cannot be applied to characterize the composition of Lig and CLig due to their poor solubility in common organic solvents. In order to detect silicon and nitrogen in lignin, X-ray fluorescence spectroscopy analysis (XRF) (Bruker AXS GMBH, Karlsruhe, Germany) was used to characterize elementals content in Lig and CLig, and the results are shown in Table 2 The CLig has much higher amount of silicon element as compared with Lig, which confirms the incorporation of silicon element caused by the reaction between reactive hygroxyl groups of Lig and silanol from hydrolysis of silane. Moreover, additional sulfur, calcium and copper elements are introduced during the process of purification and separation of lignin. Therefore, the incorporation of silicon and nitrogen elements in the lignin is confirmed by the FTIR and XRF measurements.

#### 3.1.3. Thermal Stability

**Figure 2:**
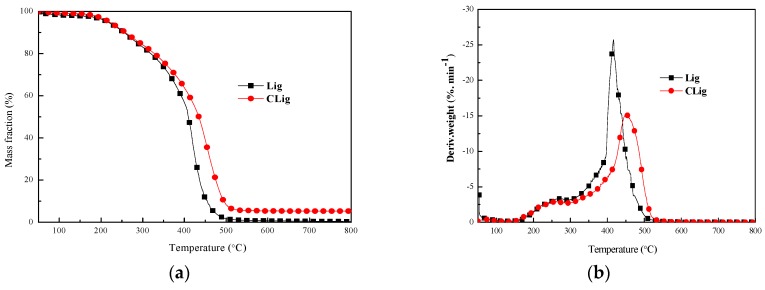
TG curves (**a**) and DTG curves (**b**) of Lig and CLig under air atmosphere.

Thermogravimetric and differential thermogravimetric curves of Lig and CLig under air atmosphere are shown in Figure 2 and the related thermogravimetric data are shown in Table 3. Two-stage thermal oxidation processes are maintained in Lig and CLig. Lig shows initial decomposition temperature (T_5%_) with 214 °C, T_max1_ with 271 °C and T_max2_ with 417 °C, and no residue is left at 800 °C. As comparison, the T_5%_ and T_max2_ temperatures of CLig are respectively enhanced by 6 °C and 35 °C, and 5% residue is left at 800 °C, although the T_max1_ temperature decreases slightly to 261 °C. Such results indicates that CLig presents better thermal stability and charring ability, which can be attributed to the incorporation of silicon and nitrogen elements to promote lignin’s charring at lower temperature. The char layer protects the underlined lignin from degradation, and leads to the higher T_max2_ and charring residue of lignin, which provide the possibility as charring agent in halogen-free flame retardant polymeric materials.

### 3.2. Flame Retardant Properties of PLA Composites

A new intumescent flame retardant (IFR) consists of APP and CLig as a charring agent, and is added in PLA to prepare PLA/IFR composites. The limiting oxygen index (LOI) and vertical burning UL-94 methods are utilized to characterize the flame retardancy of all the samples, and the testing results are shown in Table 1. As shown in Table 1, the LOI value of neat PLA is as low as 21%, meaning PLA is easily burned when exposed to fire. Incorporation alone of CLig shows weak effect on the flame retardancy of PLA. The LOI value of PLA/CLig is only 22% accompanied with UL-94 vertical burning no rating. APP shows better flame retardance effect on PLA. A high LOI value of 29% can be achieved by incorporation of 23 wt% APP into PLA, however, the UL-94 vertical testing of PLA/APP only passed V-2 rating. While IFR composed of CLig and APP can obviously enhance the flame retardancy of PLA.

**Figure 3:**
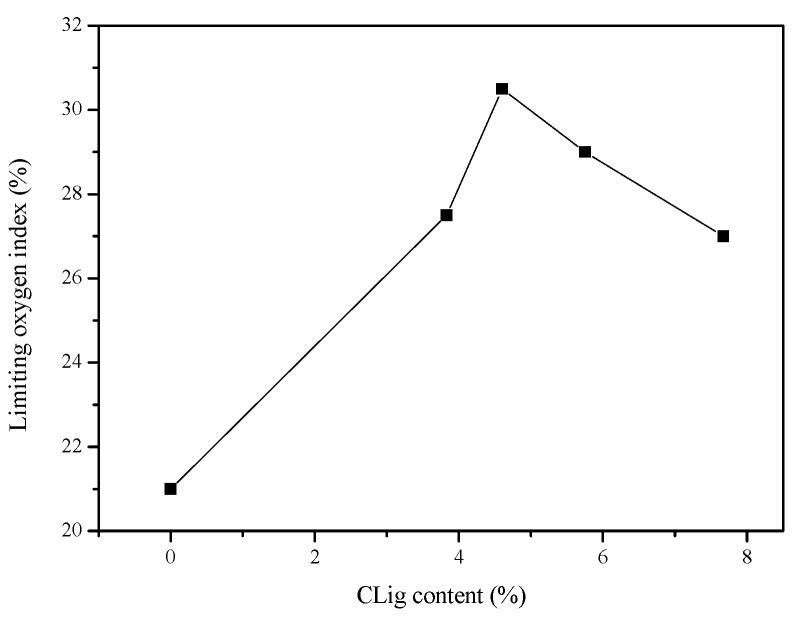
Effect of CLig content on flame retardancy of PLA/IFR composites with a total loading of 23 wt%.

Figure 3 shows the effects of Clig content on the LOI values of PLA/IFR composites with 23 wt% total loading of APP and CLig. It can be seen that the introduction of IFR significantly enhances flame retardant property of the PLA composites. With the increase of CLig content, the LOI values increase firstly, and reach the maximum value 30.5% at the 4.6% of CLig content. However, when the content of CLig was beyond 4.6%, the LOI value decreased. This could be attributed to the unmatched IFR system in which there is a suitable weight ratio among its three components to reach the optimum flame retardancy for materials. Table 1 also shows the flame retardancy of PLA containing APP and unmodified lignin (Lig). It revealed that the flame retardant properties of PLA/APP/Lig dramatically declines. This indicates that APP and CLig show much better synergistic effect on enhancing the flame retardancy of PLA than APP and Lig.

### 3.3. Combustion Behavior Analysis of PLA Composites

**Figure 4:**
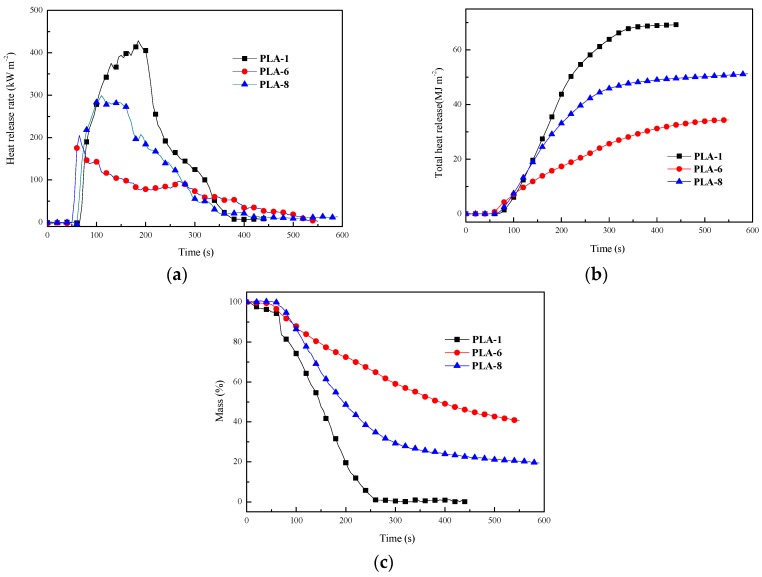
(**a**) HRR curves; (**b**) THR curves; (**c**) MLR curves of PLA and PLA composites.

Figure 4a–c illistrated heat release rate (HRR), total heat release (THR) and mass loss rate (MLR) curves, respectively, and the detailed typical parameters of pure PLA and flame retardant PLA composites are shown in Table 4 by cone calorimeter testing (CCT) at a heat flux of 35 kW/m^2^.

Table 4 shows that the time to ignition (TTIs) of the PLA composites reduce greatly compared with that of pure PLA (PLA-1), which is a typical performance of intumescent flame retardant (IFR) system [27,28]. This may be attributed to the flame retardant mechanism of IFR incorporated into PLA, decreasing the initial thermal stability of the material to form an intumescent charring layers on the surface of matrix, and hence shortening the time to ignition [22]. HRR is an important parameter used to evaluate the intensity of fires [29]. Generally, an effective flame retardant system has a lower HRR value including PHRR and average heat release rate (AHRR). The HRR curves of the samples are presented in Figure 4a. It can be seen that the pure PLA has higher PHRR and average heat release rate (Av-HRR) values, being 428.4 kW/m^2^ and 184.6 kW/m^2^, respectively. But, with the incorporation of 23 wt% loading level of flame retardant, both the PHRR and Av-HRR values of PLA obviously reduce. The PHRR values of PLA/APP/CLig (PLA-6) and PLA/APP/Lig (PLA-8) are 205.3 kW/m^2^, and 299.4 kW/m^2^, respectively, which decreases by 52.1%, and 30.1%, respectively. Meanwhile, their Av-HRR values decrease respectively to 68.7 kW/m^2^ and 96.7 kW/m^2^, showing similar change tendency. PLA-6 shows the lowest PHRR and Av-HRR values, which corresponds well with the results of LOI and UL-94 vertical testing as shown in Table 1. It may be ascribed to the good synergistic effects between APP and CLig in PLA. However, if CLig is replaced by Lig, an antagonistic effect takes up.

Figure 4b exhibits that PLA-6 has the lowest THR with 34.3 MJ/m^2^. The reason could be that incorporation of nitrogen and silicon into lignin could enhance the strength of intumescent char layer, so that it can block combustible gases and heat from penetrating the matrix during combustion, and prevent material from cracking and undergoing further degradation. The time to reach the peak of heat release rate value (TTPH) shows that the formation of intumescent charring layerneeds much less time for PLA-6, and further confirms that CLig can accelerate the charring process. This could account for the reason that PLA-6 has higher LOI value than PLA-8 mentioned above.

The mass loss rate (MLR) curves of samples are illustrated in Figure 4c. It can be seen that the mass loss rates of flame retardant PLA composites (PLA-6 and PLA-8) are significantly lower than pure PLA (PLA-1), and especially the PLA-6 presents the lowest one with highest residual mass 40.6% at the end of burning. These results indicate that for PLA-6 the char layer has good quality and higher amount during combustion due to the positive synergistic effect offered by CLig and APP.

### 3.4. Thermal Stability of Flame Retardant PLA Composites

**Figure 5:**
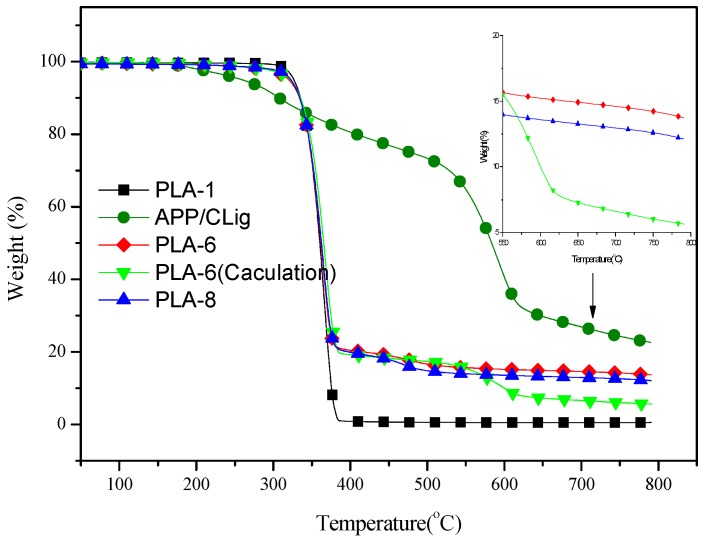
TG curves of PLA, APP/Clig, PLA/IFR composites and PLA-6(Calculation) under N_2_.

TG curves of pure PLA and flame retardant PLA composites under N_2_ atmosphere are illustrated in Figure 5 and the analysis data are shown in Table 5. Pure PLA (PLA-1) started to decompose at around 327 °C, and has few residues above 400 °C. Incorporation of flame retardant has no obvious affect on the initial thermal degradation temperature (T_5%_) of PLA although the T_5%_ shifts to a little lower temperature [30], while the residues significantly increase. Besides, for PLA-6, the residual char at high temperature is higher than that of PLA-8. This could be inferred that the existence of silicon and nitrogen elements in lignin enhances the thermal stability of flame retardant PLA at high temperature. In order to further confirm the synergistic effect between APP and CLig in PLA-6, thermal stability of APP/CLig with 4:1 weight ratio is also investigated.

The PLA-6 calculation TG curve was obtained by plotting the mass fraction of PLA-6 calculated by weight fractions of neat PLA and APP/CLig from their experimental TG curves at the mass percentage of 70% and 30% at specific temperature versus temperature, and also seen in Figure 5. No difference between the experimental curve and the calculated one below 500°C was observed, while the experimental char residues were higher than the corresponding calculated values, in which the experimental char residues at 600 °C and 800 °C reached 15.2% and 13.7% respectively, but the corresponding char residues by calculation are only 10.0% and 5.6%. This suggests that the positive synergistic effect between APP and CLig benefits to enhancement of charring performance of lignin as well as thermal stability of PLA at high temperatures, and thus the flame retardant property is improved.

### 3.5. Structure and Morphology of Char Residues

**Figure 6:**
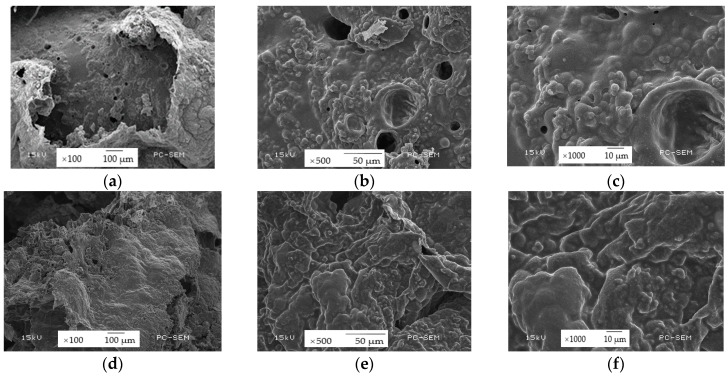
SEM micrographs of residues of PLA-8 (**a** ×100, **b** ×500, **c** ×1000) and PLA-6 (**d** ×100, **e** ×500, **f** ×1000) after cone calorimeter testing.

The char residues play a significant role in improving flame retardancy of PLA. In order to clarify the relationship between the flame retardant performance and the microstructure of char residues of flame retardant PLA composites, the investigation of combustion residues after cone calorimeter testing is conducted by SEM (Carl Zeiss Company, Jena, Germany) and Raman spectroscopy (Renishaw Company, London, UK). Figure 6 shows the SEM micrographs of char residues of PLA-6 and PLA-8 after cone calorimeter testing. As shown in Figure 6a–c, the char residue of PLA-8 displays a loosely spheroidal structure with some cavities, which is probably caused by the volatile gases generated during burning process. Thus, heat and volatiles can easily penetrate the char layers, leading to the poor fire protection [25]. While for PLA-6 with CLig, it can be seen from Figure 6d–f that there is a continuous and compact structure with few amounts of cavity on the surface. It suggests that the incorporation of CLig in flame retardant PLA boosts the formation of compact and continuous char, which can prevent heat transfer and protect the underlying polymeric substrate [31]. 

**Figure 7:**
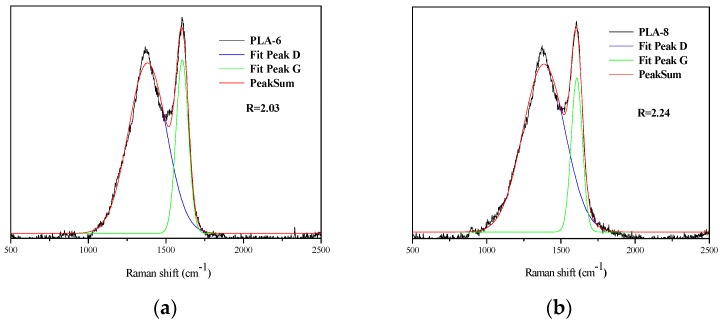
Raman spectroscopy analysis curves of char residue for PLA composites: (**a**) PLA-6 and (**b**) PLA-8.

Raman spectroscopy is an effective way to measure the hybridization state of carbon atoms in the residual char [32,33]. It can be obtained from Figure 7 that the residues of both PLA-6 and PLA-8 display two characteristic bands, located at 1583 cm^−1^ and 1359 cm^−1^, namely G-band and D-band respectively. The ratio (R) of the integral peak intensity of D band and G band, means the graphitization degree of the chars, and the higher the value of R is, the lower the graphitization degree of chars will be [34,35]. According to the integrated results, the R values of PLA-6 and PLA-8 are 2.03 and 2.24 respectively, inferring that the PLA-6 has much higher graphitization degree. These phenomena can agree with the above analysis results well.

### 3.6. Mechanical Properties of PLA Composites

Table 6 presents the tensile strength and elongation at break of pure PLA and PLA composites. Pure PLA (PLA-1) shows a tensile strength of 61.1 MPa and an elongation at break of 5.8%. Generally, the incorporation of flame retardants deteriorates the mechanical properties of polymer composites. It suggests that with the addition of APP/Lig or APP/CLig, the tensile strength of PLA composites dramatically decrease, while their elongation at break slightly changes. Meanwhile, PLA-6 has better mechanical performance than PLA-8, and its elongation at break is even higher than that of PLA-1. This may be ascribed to the weaker intra or intermolecular hydrogen bond effects among the lignin caused by modification by B201, and the improvement of lignin’s dispersion in PLA matrix [36]. Although the tensile strength of PLA-6 declines due to the introduction of APP and CLig, it can still meet the industrial standards of halogen-free flame retardant PLA composites in most conditions [31].

## 4. Conclusions

Wheat straw soda lignin containing silicon and nitrogen elements (CLig) was prepared by the reaction between lignin and γ-divinyl-3-aminopropyltriethoxysilane, and the CLig had better thermal stability compared with unmodified lignin. The CLig combined with APP was applied to obtain flame retardant PLA. APP and CLig with 4:1 mass ratio presented the optimum synergistic flame retardant effect in PLA, in which PLA passed UL-94 V-0 rating and its LOI value reached 30.5%. Compared with PLA/APP/Lig, the PHRR and THR of PLA/APP/CLig largely reduced, and the residues were raised. TGA results showed that the synergistic effect between APP and CLig could promote PLA to decompose lower temperature to form intumescent char, leading to thermal stability at higher temperature. morphology and structure analysis results of Residual char showed that the synergistic effect between CLig and APP result in forming a continuous, compact and intumescent char layer on the surface of materials during firing, and higher graphitization degree. PLA/APP/CLig had much better mechanical properties. CLig meets the sustainable development of materials, and will be a promising candidate for a charring agent in halogen-free PLA composites.

## Figures and Tables

**Table 1:** Composition of the samples and the testing results of neat PLA and PLA composites’ LOI and UL-94 vertical burning.

Sample	Component (wt%)				LOI (%)	UL-94		
	PLA	APP	CLig	Lignin		Ranking	Ignition of the Cotton	t1/t2 (s)
PLA-1	100	-	-	-	21	NR	Yes	>30
PLA-2	77	23	-	-	29	V-2	Yes	11/0
PLA-3	77	-	23	-	22	NR	Yes	>30
PLA-4	77	15.33	7.67	-	27	V-1	No	9/2
PLA-5	77	17.25	5.75	-	29	V0	No	1/0
PLA-6	77	18.40	4.60	-	30.5	V0	No	0/1
PLA-7	77	19.17	3.83	-	27.5	V2	Yes	12/0
PLA-8	77	18.40	-	4.60	25	V0	No	2/3

**Table 2:** Element contents of Lig and CLig obtained by XRF.

	Elements	O/%	Si/%	S/%	Fe/%	Ca/%	Cu/%
Samples							
Lig		91.63	2.58	4.19	1.28	0.18	0.14
CLig		79.85	14.33	4.07	1.33	0.22	0.20

**Table 3:** Related thermal degradation parameters of Lig and CLig under air atmosphere.

Samples	T_5%_ (°C)	T_50%_ (°C)	T_max (%)_		Residual (%) 800 °C
			T_max1_	T_max2_	
Lig	214	409	271	417	0
CLig	220	435	261	452	5

**Table 4:** Cone calorimeter testing data of PLA and PLA composites.

Sample	TTI (s)	PHRR (kW/m^2^)	Av-HRR (kW/m^2^)	TTPH (s)	THR (MJ/m^2^)	Residual Mass (%)
PLA-1	64	428.4	184.6	185	69.2	0
PLA-6	48	205.3	68.7	65	34.3	40.6
PLA-8	58	299.4	96.7	110	51.3	19.5

**Table 5:** Thermal degradation parameters data of PLA and PLA composites under N_2._

Samples	T_5%_ (°C)	T_max_ (°C)	Residue (%)					
			600 °C		700 °C		800 °C	
			Exp.	Cal.	Exp.	Cal.	Exp.	Cal.
APP	302	593	33.2	-	20.4	-	16.3	-
CLig	199	356	41.5	-	39.3	-	38.6	-
APP/CLig(4:1)	259	593	40.7	34.7	26.9	24.1	22.6	20.7
PLA-1	327	366	0.5	-	0.5	-	0.5	-
PLA-6	320	375	15.2	10.0	14.6	6.6	13.7	5.6
PLA-8	323	365	13.6	-	13.0	-	12.1	-

**Table 6:** Mechanical properties of PLA and PLA/IFR composites.

Sample	Tensile Strength (MPa)	Elongation at Break (%)
PLA1	61.1 ± 3.1	5.8 ± 0.2
PLA6	41.2 ± 2.1	6.6 ± 0.3
PLA8	29.5 ± 1.5	5.2 ± 0.2
